# Barriers and facilitators for cervical cancer screening among adolescents and young people: a systematic review

**DOI:** 10.1186/s12905-021-01264-x

**Published:** 2021-03-23

**Authors:** Abirami Kirubarajan, Shannon Leung, Xinglin Li, Matthew Yau, Mara Sobel

**Affiliations:** 1 Faculty of Medicine, University of Toronto, 1 Kings College Circle, Medical Science Building, Toronto, ON M5S 1A8 Canada; 2 Institute of Health Policy Management and Evaluation, University of Toronto, Toronto, ON Canada; 3 Department of Obstetrics and Gynecology, Sinai Health System, Toronto, ON Canada

**Keywords:** Cervical cancer, Young people, Adolescent, Screening, Pap smear, HPV, Teenager, Youth, pap test

## Abstract

**Background:**

Though cervical cancer is one of the leading causes of cancer-related death globally, its incidence is nearly entirely preventable. Young people have been an international priority for screening as this population has historically been under-screened. However, in both high-income and low-income countries, young people have not been screened appropriately according to country-specific guidelines. The aim of this systematic review was to systematically characterize the existing literature on barriers and facilitators for cervical cancer screening (CCS) among adolescents and young people globally.

**Methods:**

We conducted a systematic review following PRISMA guidelines of three key databases: Medline-OVID, EMBASE, and CINAHL. Supplementary searches were done through ClinicialTrials.Gov and Scopus. Databases were examined from 1946 until the date of our literature searches on March 12th 2020. We only examined original, peer-reviewed literature. Articles were excluded if they did not specifically discuss CCS, were not specific to individuals under the age of 35, or did not report outcomes or evaluation. All screening, extraction, and synthesis was completed in duplicate with two independent reviewers. Outcomes were summarized descriptively. Risk of bias for individual studies was graded using an adapted rating scale based on the Risk of Bias Instrument for Cross-Sectional Surveys of Attitudes and Practices.

**Results:**

Of the 2177 original database citations, we included 36 studies that met inclusion criteria. The 36 studies included a total of 14,362 participants, and around half (17/36, 47.2%) of studies specifically targeted students. The majority of studies (31/36, 86.1%) discussed barriers and facilitators to Pap testing specifically, while one study analyzed self-sampling (1/36, 2.8%), one study targeted HPV DNA testing (1/36, 2.8%), and the remainder (4/36, 11.1%) were not specified. Our systematic review found that there are three large categories of barriers for young people: lack of knowledge/awareness, negative perceptions of the test, and systemic barriers to testing. Facilitators included stronger relationships with healthcare providers, social norms, support from family, and self-efficacy.

**Conclusion:**

There are unique barriers and facilitators that affect CCS rates in adolescents and young people. Health systems and healthcare providers worldwide should address the challenges for this unique population.

## Background

Cervical cancer is the second most common malignancy among women worldwide, with over 600,000 new cases and 300,000 deaths annually [1–3]. The disease is frequently caused by the human papillomavirus (HPV), which is sexually transmitted [1]. Though cervical cancer is one of the leading causes of death globally, its incidence is nearly entirely preventable [4]. Cervical cancer screening (CCS) and HPV vaccination programs have significantly reduced the mortality of cervical cancer in North America and Europe through secondary prevention. Screening techniques include Papanicolau tests (also known as Pap smears), liquid-based cytology, HPV DNA testing, and visual inspection with acetic acid [5, 6]. Through timely CCS, patients can obtain referrals to colopscopy and receive definitive treatment for abnormal cervical cells or malignancy. Despite the proven effectiveness of CCS, there are numerous barriers to uptake, particularly in low-income countries [7].

Young people have been a particular area of research focus, due to the preventive benefits of screening from a younger age, increased likelihood of lifelong testing, and setting of new cultural norms [8–11]. In both high-income and low-income countries, young people have not been screened appropriately according to country-specific guidelines and in many countries, screening rates for this age-group have even dropped [12–16].

As a result, numerous interventions have been posited to increase CCS among young people [8]. However, there has not yet been a systematic assessment of the barriers and facilitators that determine uptake among this age-group. This information would be useful in designing targeted and efficacious interventions. The aim of this systematic review was to systematically characterize the global literature on barriers and facilitators for CCS among young people.

## Methods

This systematic review was conducted and reported according to the standards and guidelines established in the Preferred Reporting Items for Systematic Reviews and Meta-Analysis (PRISMA), in addition to the fourth edition of the Joanna Briggs Institute Reviewer’s Manual [17, 18].

### Search strategy

We conducted a systematic literature search of three key databases: Medline-OVID, EMBASE, and CINAHL. Supplementary searches were done through ClinialTrials.gov and Scopus.

Our search criteria included broad keywords and subject headings in order to maximize sensitivity. We did not apply any filters on the basis of language or country of origin. Our search strategy is included in Table 1.

**Table 1:** Search strategy

#	Searches
1	VAGINAL SMEARS/
2	(vagina* AND smear*).ti,ab
3	(pap AND test). ti,ab
4	cytology.ti,ab
5	(pap AND smear). ti,ab
6	(cervical adj2 (smear OR screen*)).ti,ab
7	(papanicolaou adj2 (smear OR test*)).ti,ab
8	1 OR 2 OR 3 OR 4 OR 5 OR 6 OR 7
9	(youth* or adolescen* or (young adj2 (adult* or person* or individual* or people* or population* or wom#n)) or youngster* or college* or university*).ti,ab. or adolescent/ or young adult/
10	(barrier* OR facilitator* OR perception* OR perspective* OR utilization* OR view*).ti,ab
11	8 AND 9 AND 10

Database: Ovid MEDLINE: Epub Ahead of Print, In-Process & Other Non-Indexed Citations, Ovid MEDLINE® Daily and Ovid MEDLINE®

1946—March 12 2020

*Adapted for EMBASE and CINAHL*

### Selection criteria

We defined the study population as individuals under the age of 35. The maximum age was determined based on previous literature regarding young people and CCS [8, 12]. There was no cut-off for a minimum age, as we were interested to examine the earliest age at which adolescents or young adults were screened.We only examined original, peer-reviewed literature. Databases were examined from inception until the date of our literature searches on March 12th 2020. Published conference posters, papers, and abstracts were eligible for inclusion. Articles were excluded if they did not specifically discuss CCS, were not specific to young people under the age of 35 (as reported in the title or abstract), or did not report outcomes or evaluation. Studies with transgender men, cisgender women, and intersex people with cervixes were eligible for inclusion. Eligibility criteria are outlined in Table 2.

**Table 2:** Eligibility criteria

*Population*: Young people (defined as 35 years of age or under) with cervixes of any country worldwide	
*Intervention*: Any assessment of patient-reported barriers and facilitators related to cervical cancer screening	
*Comparator*: N/A	
*Outcomes*: Any outcome reported in the literature (qualitative or quantitative)	

### Data extraction and quality assessment

All steps of the systematic review were performed in duplicate. Study selection was completed by two independent, parallel reviewers (AK, SL) for both title and abstract screening as well as full-text screening. Data extraction was performed by two investigators (AK, SL), with a third (XL) resolving discrepancies. Risk of bias for individual studies was graded using an adapted rating scale based on the Risk of Bias Instrument for Cross-Sectional Surveys of Attitudes and Practices [19].

### Analysis

Outcomes were summarized descriptively via thematic analysis. Thematic analysis was decided via consensus approach by the two reviewers (AK, SL). We did not register our systematic review to allow for iterative categorization. In addition, it was decided a priori that a meta-analysis would not be suitable for this review, due to the heterogeneity of the included articles.

## Results

**Fig. 1:**
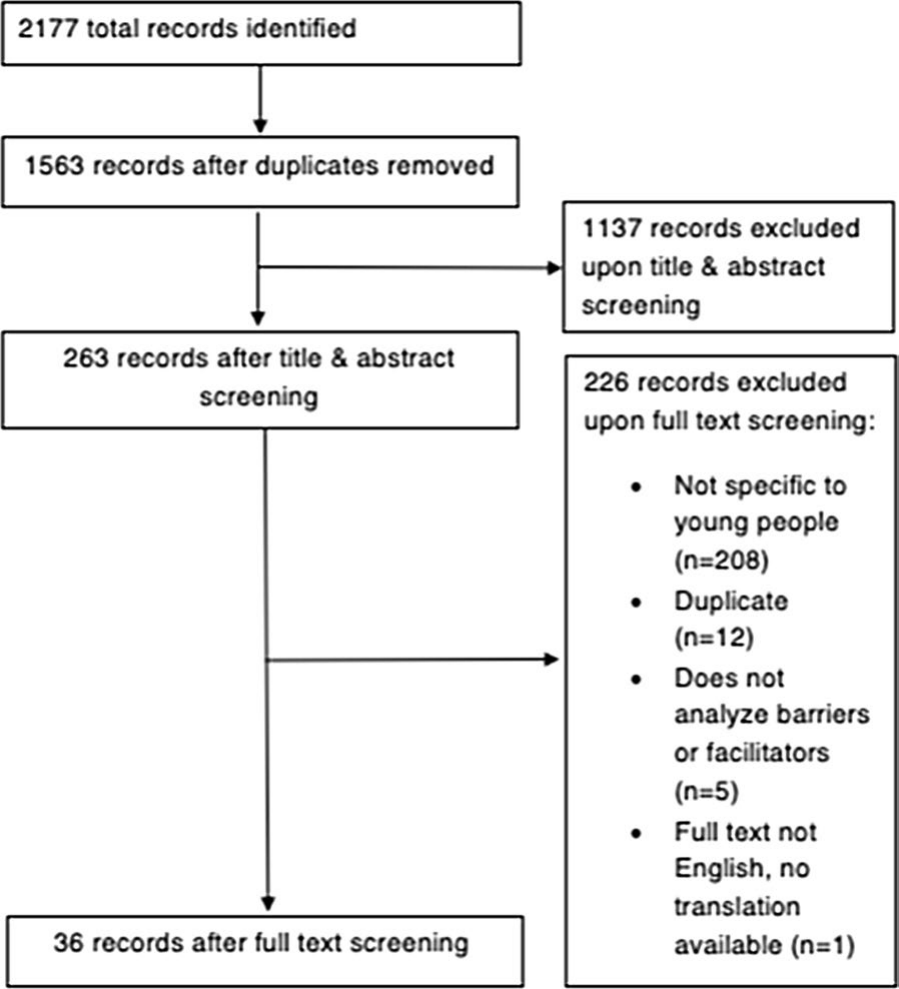
PRISMA diagram

Results of the study screening process are available in the PRISMA diagram in Fig. 1. Of the 2177 original database citations, 1563 records remained after duplicates were removed. After title and abstract screening, 226 were eligible for full-text evaluation. After a hand-search of relevant journals and citations, no additional studies were added. Of the 226 full-text articles, a total of 36 were included in the systematic review.

Inter-rater agreement for study screening for titles and abstracts was 94.54% with a kappa of 0.79. Inter-rater agreement for full-text screening was 96.2% with a kappa of 0.84, indicating substantial agreement.

### Article characteristics

The study locations were highly diverse and featured a wide spread across Africa (8/36, 22.2%), Asia (8/36, 22.2%), North America (11/36, 30.6%), South America (2/36, 5.6%), Australia (1/36, 2.8%), Europe (6/36, 16.7%). High-income countries included the United States, Canada, Denmark, Sweden, Japan, Korea, Australia, and the United Kingdom. Low- and middle-income countries included Brazil, China, Ghana, Malaysia, Nigeria, Oman, and Saudi Arabia.

The included study designs were largely qualitative and observational. The majority (25/36, 69.4%) included either surveys or questionnaires, with the remainder including either focus groups (7/36, 19.4%) or interviews (2/36, 5.6%). All studies were graded as medium risk for bias using the Risk of Bias Instrument for Cross-Sectional Surveys of Attitudes and Practices.

The 36 studies included a total of 14,362 participants, and around half (17/36, 47.2%) of studies specifically targeted students.

The majority of studies (31/36, 86.1%) discussed barriers and facilitators to Pap testing specifically, while one study analyzed self-sampling (1/36, 2.8%), one study targeted HPV DNA testing (1/36, 2.8%), and the remainder (4/36, 11.1%) were not specified.

Details of the included studies are provided in Table 3.

**Table 3:** Details of included studies

References; Country	Study design; graded Risk of Bias	Sample size; population details	Average age (SD)	Type of screening	Barriers	Facilitators
Abotchie and Shokar [20]; Ghana	Cross-sectional survey; medium risk	157 university students	NR (age range 20–35, most between 21 and 25)	Pap smear	Low knowledge, low awareness, concern regarding partner disapproval, cost, time constraints, embarrassment, perceived not susceptible, fear of virginity loss	Knowledge of benefits of screening, perception of severity of disease
Agboeze et al. [21]; Nigeria (Abstract only)	Cross-sectional survey; medium risk	234 female students	22 (SD 3)	Pap smear	Low awareness, low accessibility, fear of cancer diagnosis	NR
Akujobi et al. [22]; Nigeria (Abstract only)	Cross-sectional survey; medium risk	220 third and fourth year female science students	23.8 (SD NR)	Pap smear	Lack of knowledge, lack of awareness about importance of screening, lack of awareness about where services are attainable	NR
Al-Naggar et al. [23]; Malaysia	Cross-sectional survey; medium risk	287 female university students	20.9 (SD 1.89)	Pap smear	Fear of intimate nature of examination, HCP did not recommend/educate, lack of awareness of screening sites, cost, fear of virginity loss, embarrassment, fear of pain and discomfort	NR
Al-Shaikh et al. [24]; Saudi Arabia	Cross-sectional survey; medium risk	1400 students studying health fields	20.4 (SD 1.3)	Pap smear	Lack of awareness, perception of low sensitivity of test, misconception of serious complications of test	NR
Albuquerque et al. [25]; Brazil	Cross-sectional survey; medium risk	493 women,, young women (< 20) n = 64	35.4 (SD NR)	Pap smear	Low knowledge and awareness	NR
Alwahaibi et al. [26]; Oman	Cross-sectional survey; medium risk	494: 204 patients, 133 staff, 157 students	NR (students all age 20–30)	Pap smear	Low knowledge, lack of awareness, physician gender, uncertainty of reliability of Pap smears	Belief of test allowing successful prevention and treatment for cancer
Annan et al. [27]; Ghana	Cross-sectional survey; medium risk	200 female university students	20.4 (SD 1.96)	Unspecified	NR	Cervical cancer knowledge, perceived susceptibility, perceived deadliness, perceived benefits of screening
Ayinde et al. [28]; Nigeria	Cross-sectional survey; medium risk	421 undergraduate students	23.6 (SD 3.6)	Pap smear	Lack of awareness	Increased knowledge and awareness
Bigaard et al. [29]; Denmark (Abstract only)	Qualitative focus groups (cross-sectional); medium risk	Sample size not reported in abstract; HPV vaccinated women	NR (age range 23–29)	Pap smear	Lack of knowledge, perceived not susceptible (test not relevant to them)	NR
Binka et al. [30]; Ghana	Cross-sectional survey; medium risk	410 female students	NR (83% < 29 years; 17% > 30)	Unspecified	Lack of awareness	NR
Black et al. [31]; Canada	Cross-sectional focus groups; medium risk	80 women	NR (98% between age 20 and 29)	Pap smear	Discontinuity of care from HCP after moving away for work/school, difficulty finding female HCP, fear of discomfort and invasiveness of test, lack of awareness, lack of time	Reminders, email from HCP, linking testing to renewal appointment for oral contraceptives, assistance with finding HCP, longitudinal relationship with HCP or clinic, education, testing by female HCP, increased convenience (e.g. clinic on campus)
Blomberg et al. [32]; Sweden	Cross-sectional focus groups; medium risk	138 women from Stockholm cervical cancer screening registry	30 (SD 0)	Pap smear	Fear of pain and discomfort	Letter of invitation, flexibility in timing and location, choice of HCP, having test done with other exams, social marketing on importance of screening, cost-free testing
Blomberg et al. [33]; Sweden	Cross-sectional focus groups; medium risk	38 women from Cervical Cancer Screening administrative registry	30 (SD 0)	Pap smear	Lack of knowledge, lack of awareness, perceived not susceptible	Existing relationship with clinic
Byrd et al. [34]; United States	Cross-sectional survey; medium risk	200 women	21 (SD NR)	Pap smear	Embarrassment, pain, embarrassment, fear of virginity loss, lack of awareness of where to be tested, fear of partner disapproval, misconception (only women who have had babies) need to be tested, fear of testing perceived as sexual activity	NR
Duffet-Leger et al. [35]; Canada	Cross-sectional survey; medium risk	1041 university students	20.7 (SD 1.77)	Pap smear	NR	Beliefs/behaviours of friends and family, sense of empowerment about getting Pap test
Head and Cohen [36]; United States	Cross-sectional individual and group interviews; medium risk	19 women	NR (median age of 20)	Pap smear	Negative past experiences, limited choice in HCP, fear of parental disapproval, cost, low accessibility, lack of privacy in small community (e.g. running into someone they know at the clinic)	Encouragement/support from mother
Hobbs [37]; United States (Abstract only)	Qualitative focus groups; medium risk	15 sexually active adolescents	18.7 (SD NR)	Pap smear	Lack of knowledge, fear of pain, embarrassment, fear of result, fear of unknown, perceived invincibility, HCP characteristics, fear of parents finding out, cost, lack of time, transportation	Education, trusting relationship with HCP, HCPs able to communicate well and put patients at ease, assured confidentiality, school programs, shorter wait times, telephone/email reminders, provision of babysitting or transportation, expanded clinic hours, having someone answer the phone when patients book appointments
Hoque [38], South Africa	Questionnaire; medium risk	169 full-time undergraduate students	20.81 (SD 1.7)	Pap smear	Low knowledge, fear of procedure, not currently experiencing symptoms	NR
Hoque [39], South Africa (Abstract only)	Questionnaire; medium risk	440 university students	20.39 (SD 1.71)	Pap smear	Low knowledge	High self-efficacy
Jayasinghe [40], Australia	Online survey; medium risk	149 women	23.2 (SD 2.1)	HPV DNA testing	Repetitive screening frequency	Public perceptions, national guidelines, gynecologist beliefs
Jubelirer [41]; United States	Questionnaire; medium risk	279 tenth-grade girls	15.3 (SD NR)	Pap smear	Embarrassment, pain, fear of cancer, confidentiality, cost	NR
Kahn [42], United States	Semi-structured interview; medium risk	15 adolescents	18.7 (SD 1.9)	Pap smear	Pain, embarrassment, fear of cancer, denial, poor HCP relationship, low knowledge, misinformation	Education, better HCP relationships, telephone and written reminders
Kahn [28], United States	Questionnaire; medium risk	490 adolescents and young girls at pap smear follow-up clinics	18.2 (SD NR)	Pap smear	Pain, inconsistent HCP, embarrassment, lack of HFP communication, lack of time, lack of transportation	Reminders, perceived control, perceived susceptibility
Kaneko [43], Japan	Online survey; medium risk	700 unmarried women	26.0 (SD NR)	Pap smear	Male physician	Free coupon for screening, perceived susceptibility
Kim [44], Korea (Abstract only)	Questionnaire; medium risk	303 unmarried female university students	22.4 (SD 2.0)	Pap smear	Low knowledge/awareness	NR
Kim [45], Korea (Abstract only)	Online questionnaire; medium risk	124 unmarried university students	NR	Pap smear	Unsure of effectiveness, low awareness	Subjective norm
Langille [46], Canada	Questionnaire; medium risk	1090 high school adolescents	16.6 (SD 0.1)	Pap smear	Lack of discussion with HCP, no physician, rural area	Education from HCP
Lee [47], United States	Focus group; medium risk	16 young Korean immigrant women	26 (SD NR)	Pap smear	Low knowledge, culture-specific barriers, low accessibility to healthcare, embarrassment, fear of virginity loss, stigma around visiting gynecologist clinic, language barriers, low health literacy	NR
Lorenzi [39],Brazil	Survey; medium risk	33 women with abnormal pap smears	NR (aged < 29)	Self-sampling	Embarrassment, pain	Easy to use, easy to understand, practicality
Najem [48], United States	Survey; medium risk	3343 inner city high school girls	NR (aged 13 and up)	Pap smear	HCP did not recommend, lack of awareness, cost, unaware of location, perceived not susceptible, time constraints, believed test was not accurate, embarrassment, time constraint	Family role models
Ogbonna [49], United Kingdom	Survey; medium risk	186 UK university students from Sub-Saharan Africa	NR (majority between age 18 and 24)	Pap smear	Low knowledge, misconceptions (such as around circumcision), cultural barriers, stigma	Perception of deadliness
Okoeki [50], United Kingdom	Semi-structured interviews, focus group; medium risk	24 young women	NR (age range 25–34)	Pap smear	Low awareness, embarassment, anxiety, association with sex, intimate, cultural barriers, fear of cancer, invasiveness	Education on sensitivity, education, alternative screening methods (self-sampling)
Oshima [51], Japan	Focus group; medium risk	15 university students	NR (age 20–22)	Pap smear	Lack of knowledge,misconceptions, lack of motivation, reluctance to visit gynecologist (embarrassment, stigma, invasiveness)	Media information, norms of family and friends, diagnosis within their family, education
Pan [52], China	Survey; medium risk	1878 medical students	20.8 (SD 1.3)	Unspecified	Side effects, inadequate information, low knowledge, stigma around premarital sex	High knowledge
Waller [53], United Kingdom	Interview, focus group; medium risk	27 young women	NR (age range between 25 and 34 for young women)	Unspecified	Lack of access to HCP (mobility, lack of consistency), fewer reminders, time constraints, lack of peer pressure, low perceived low perceptibility, menstrual cycle timing, low knowledge, pain, uncomfortable comments, apathy	Awareness, media coverage

### Analysis of notable barriers

There were numerous barriers cited by the young people and adolescents regarding CCS. The barriers can be grouped into three large groups: lack of knowledge/awareness, negative perceptions of testing, and practical barriers.

(i) Lack of Knowledge/Awareness

Most notably, 26/36 (72.2%) of studies reported lack of knowledge or awareness in young people regarding cervical cancer prevention. Three studies [23, 46, 48] noted lack of physician recommendation, while one noted gaps in health literacy of the participants [47].

Misinformation included beliefs that young people were not susceptible in 4/36 studies [20, 32, 48, 53], that screening was not necessary if not currently experiencing symptoms [38], and that the Pap test was not effective/reliable for screening cervical cancer [26, 45]. Other misconceptions included that a cisgender male partner’s circumcision prevented their need for CCS [49]. In four studies, there was a fear that pelvic exams could “take one’s virginity,” reported in Ghana, Malaysia, and the United States [20, 23, 34, 47].

(ii) Negative Perceptions of Testing

A large number of young people had fears and anxieties regarding the screening experience. Ten studies cited a fear of pain/discomfort during Pap smears, with 13/36 studies noting embarrassment of the intimate examination. Three studies noted the invasiveness of the procedure being of particular concern [31, 50, 51]. Male physicians were also noted to impede motivation for screening in two studies [31, 43].

Stigma around cervical cancer was noted in 4 studies [47, 49, 51, 52], with two of the studies reporting stigma around the general act of visiting a gynecologist’s office [47, 51]. Confidentiality was a concern noted in three studies [36, 37, 41], with two specifically noting privacy from parents [36, 37].

Two studies discussed fear of side effects or complications from screening [24, 52]. Five studies discussed fear regarding potential diagnosis of cancer as a barrier to screening [21, 37, 41, 42, 50].

(iii) Systemic Barriers on Organizational Level

There were a number of systemic barriers noted to accessing CCS.

Six studies discussed low accessibility to services [21, 31, 36, 46, 47, 53]. Participants reported difficulties in finding a consistent healthcare provider, especially after moving away for work or school [31, 53]. Difficulties were also noted in rural areas with only a single provider [20] or locations with reduced access to female physicians [31].

Transportation was noted as a barrier in two studies [37, 54]. Cost of screening services and financial constraints were noted as a barrier in six studies [20, 23, 36, 37, 41, 48], with two studies located in lower income countries (Ghana, Malaysia) and the remainder in the United States.

Time constraints were cited in three studies [20, 48, 53]. One study noted that participants preferred to schedule their appointments according to their menstrual cycle, which posed further limitations [53].

### Analysis of notable facilitators

Many studies discussed facilitators and interventions that encouraged young people to undergo CCS. Increased knowledge and awareness were noted in twelve studies [20, 26–28, 37, 40, 42, 49–53]. Specific points of knowledge included severity of disease [20, 49], as well as the understanding that the test could allow successful prevention and treatment of cancer [26]. High self-efficacy and perceived control/empowerment about health was a facilitator in three studies [54, 55, 35].

Trusting and longitudinal relationships with their healthcare providers were noted as facilitators in four studies [31, 32, 37, 42], as was choice of healthcare provider specifically [32] or testing by a female physician [31]. Hobbs et al. [37] specifically noted that physicians who were able to communicate well and put patients at ease acted as a facilitator. Alternative methods of screening such as self-sampling were noted as a facilitator of CCS to avoid perceived invasiveness [50, 39].

Social norms and public perceptions, including if friends and family members received testing, was noted as a facilitator [35, 36, 40, 45, 48, 51]. A diagnosis of cervical cancer in the family was noted as a motivation for undergoing screening [51], as well as support or encouragement from one’s mother specifically [36]. Media coverage was noted to encourage participation in CCS, particularly if involving celebrities or public figures [50, 53].

Facilitators to improve the logistical barriers of cervical cancer were analyzed. Five studies noted either telephone or written reminders would be helpful for patients [31, 32, 37, 42, 54]. To address the time constraints of patients, Black et al. [31] and Blomberg et al. [32] noted that cervical screening could be linked with appointments for prescription renewals or other examinations. In addition, Blomberg et al. [32] suggested flexibility in time and location of screening, including options such as screening available on college campuses. Shorter wait-times, expanded clinic hours, and having someone pick up the phone when patients book appointments were noted as practical options by Hobbs et al. [37]. Cost-free services, provision of babysitting services, and arranged transportation were also suggested [32, 37, 43].

## Discussion

### Main findings

Our study is the first systematic review of barriers and facilitators to CCS specifically for young people and adolescents under the age of 35. While there have been calls to action regarding this topic, it has been difficult to characterize the breadth of young people’s perspectives regarding screening. Our 36 included studies had a diverse spread of country locations across low-, middle- and high-income countries in addition to a range of study populations. Barriers encompassed three groups: lack of knowledge/awareness, negative perceptions of the test, and systemic barriers to testing on an organizational level. Facilitators included stronger relationships with healthcare providers, social norms, support from family, and self-efficacy.

Our results support the current literature base regarding the uptake of CCS in young people. Young people face unique barriers and facilitators in comparison to older groups, necessitating age-specific interventions. Our studies highlighted age-specific barriers such as concern about privacy from parents, transportation difficulties, and continuity of care after moving away for school. In addition, as this is typically the first invasive procedure that young people undergo, there were concerns about pain, discomfort, and the intimacy of the pelvic exam. The young people who participated in these studies had helpful suggestions regarding age-specific interventions, such as emailed reminders in comparison to written reminders, or screening provided on college campuses. Our literature also aligns with the greater research base regarding young people and low preventative service use in general, as many young people do not have a consistent family physician [56, 57]. As such, other studies have also noted that age is a consideration for cancer screening beliefs or adherence to cancer screening programs [58, 59].

When comparing results internationally, we noticed that many themes were universally represented across income levels. There were accessibility concerns, cost concerns, and knowledge gaps in both lower and higher income countries. However, it is important to note that screening rates differ across the globe, and even within the same country for lower income and minority populations. As financial constraints were cited as a frequent barrier in our included studies, it is not surprising that people from lower socioeconomic backgrounds have lower screening rates [60, 61]. In addition, people from minority populations may have more strained relationships with the health system due to discrimination, lack of cultural competence, and the historic failure of medical systems to be equitable towards minority groups [62, 63]. This is particularly relevant to cervical screening, as the patient’s individual relationship with the health system was noted as an important barrier or facilitator towards screening. To increase cervical screening rates, it is important that we improve health system interactions overall to be more equitable.

Additionally, we noted that cultural barriers were discussed in several studies, including sex-negative beliefs [47, 49, 50, 52]. Several studies highlighted a fear of hymen breakage with the pelvic exam, which has the societal stigma against virginity loss [20, 23, 34, 47]. This concept was not only studied in Asia and Africa, but also included two studies from the United States [34, 47]. It is important to educate about the concept of virginity as a social construct and improve sexual education. In higher-income countries, language barriers, health literacy, and cultural beliefs were also noted as barriers among recent immigrants. Recent literature has shown that the “healthy immigrant effect” tends to taper off after several decades in a new country, with immigrants at higher risk of poor health outcomes and underuse of health services [64, 65]. Specific to cervical cancer, immigrant and minority populations in developed countries are at higher risk, often due to low screening rates [66, 67]. Thus, interventions that target cervical screening uptake should have an intersectional approach in addressing these issues, rather than a “one size fits all” approach. Finally, as many participants expressed a fear of the speculum examination, it is important that both medical trainees and physicians are adequately trained regarding patient comfort during speculum exams, potentially through interventions such as gynecological teaching associates or standardized patients.

### Strengths and limitations

Strengths of our review include our systematic search of multiple databases using broad search criteria to maximize findings. Studies were not excluded by basis of date of publication, country of origin, or language of origin. To capture the full breadth of explored research, conference abstracts were included. Our two parallel reviewers had high inter-rater agreement. We also included age ranges up to 35 years, based on previous literature. Limitations include that this review included qualitative studies that may have been limited by selection and publication bias, particularly for lower-income countries that may not be well-represented in academic research. Studies were also only included if the title and/or abstract explicitly discussed the age cut-off of young people, which may have inadvertently excluded relevant studies. As with any systematic review, there is a risk that relevant studies may have been excluded, despite our efforts to maximize our search's sensitivity. In addition, CCS guidelines (e.g. age to start screening) may differ depending on country, which may limit generalizability of results. Survey and interview results may also be compromised by reporting bias, if study participants are potentially embarrassed to discuss barriers or facilitators. More rigorous and systematic research with an equity-focused lens is recommended to generalize results to different populations and obtain higher quality information.

### Next steps and implications for care

Further research is required to characterize which interventions are the most effective for different age groups, including a diverse range of ethnicities, sexual orientations, educational backgrounds, and income levels. Future studies may also wish to consider other factors in awareness, such as the date of implementation of the CCS program or the presence of an HPV vaccination program. Moreover, we were surprised that none of the studies specifically targetted the beliefs of non-heterosexual or non-cisnormative participants, as this has been documented as a growing public health concern and source of misinformation [68–71]. Studies regarding transgender men were eligible for study inclusion, but yielded no search results based on our protocol as the identified studies were not specific to youth or young people. Further specific investigation is required to understand this topic, from both the perspective of the patient and the physician.

Our results were encouraging regarding potential solutions for improving CCS uptake. While some barriers such as fear of cancer diagnosis or longstanding cultural beliefs are difficult to address, other barriers offer feasible solutions. Younger people may have less control in their lives regarding transportation and scheduling, particularly when coordinating with parents, babysitting siblings, or school schedules. It was remarkable how many small changes, such as written reminders, pamphlets, or linking screening with other appointments, were noted to act as facilitators. In addition, multiple misconceptions about CCS still persist, such as the belief that screening is only required if a patient is experiencing symptoms. Our studies suggested the effectiveness of awareness campaigns that are specifically aimed at younger people. Campaigns targeting parents may also be important as studies noted that parental support was a facilitator for screening. We recommend further research on interventions, particularly educational resources such as information leaflets.

Through addressing the above barriers and facilitators, health systems worldwide can hopefully address the much-touted goal of zero preventable deaths from cervical cancer. Young people who undergo screening are more likely to continue the practice as a lifelong habit as well as later recommend the practice to their children and peers [8–11]. They are also more likely to espouse positive beliefs about the health system, sex-positive beliefs, and regain empowerment regarding their health [72, 73]. As such, every young person who is screened offers a chance of strengthening a community around reproductive health.

## Conclusion

Our comprehensive systematic review found that there are three large categories of barriers for young people: lack of knowledge/awareness, negative perceptions of the test, and systemic barriers to testing. Facilitators included stronger relationships with healthcare providers, social norms, support from family, and self-efficacy. Health systems worldwide should address the above barriers and facilitators to increase CCS rates in young people.

## Data Availability

The datasets used and/or analysed during the current study are available from the corresponding author on reasonable request.

## Abbreviations

CCS: Cervical cancer screening
HPV: Human papillomavirus
PRISMA: Preferred Reporting Items for Systematic Reviews and Meta-Analysis

## References

[1] Behtash N, Mehrdad N (2006). Cervical cancer: screening and prevention. Asian Pac J Cancer Prev.

[2] GLOBOCAN 2020: cancer incidence, mortality and prevalence worldwide. IARC Press. Available from https://gco.iarc.fr/today/data/factsheets/cancers/23-Cervix-uteri-fact-sheet.pdf

[3] World Health Organization (2009). Initiative for vaccines research team of the department of immunization, vaccines and biologicals. Available at: http://www.who.int/vaccinesdocuments/. Accessed March 2020.

[4] Arbyn M, Weiderpass E, Bruni L, de Sanjosé S, Saraiya M, Ferlay J, Bray F (2020). Estimates of incidence and mortality of cervical cancer in 2018: a worldwide analysis. Lancet Global Health.

[5] Sankaranarayanan R, Gaffikin L, Jacob M, Sellors J, Robles S (2005). A critical assessment of screening methods for cervical neoplasia. Int J Gynecol Obstetr.

[6] Nuovo J, Melnikow J, Howell L (2001). New tests for cervical cancer screening. Am Fam Physician.

[7] Islam RM, Billah B, Hossain MN, Oldroyd J (2017). Barriers to cervical cancer and breast cancer screening uptake in low-income and middle-income countries: a systematic review. Asian Pacific J Cancer Prev APJCP.

[8] Albrow R, Blomberg K, Kitchener H, Brabin L, Patnick J, Tishelman C, Törnberg S, Sparén P, Widmark C (2014). Interventions to improve cervical cancer screening uptake amongst young women: a systematic review. Acta Oncol.

[9] Peto J, Gilham C, Fletcher O, Matthews F (2004). The cervical cancer screening epidemic that screening has prevented in the UK. Lancet.

[10] Jepson R, Clegg A, Forbes C, Lewis R, Sowden A, Kleijnen J. The determinants of screening uptake and interventions for increasing uptake: A systematic review. Health Technol Assess 2000;4:i–vii, 1–133. 10984843

[11] Ferrer HB, Trotter C, Hickman M, Audrey S (2014). Barriers and facilitators to HPV vaccination of young women in high-income countries: a qualitative systematic review and evidence synthesis. BMC Public Health.

[12] Bradley J, Risi L, Denny L (2004). Widening the cervical cancer screening net in a South African township: who are the underserved?. Health Care Women Int.

[13] Lancucki L, Fender M, Koukari A, Lynge E, Mai V, Mancini E (2010). A fall-off in cervical screening coverage of younger women in developed countries. J Med Screen.

[14] Llorca J, Rodriguez-Cundin P, Dierssen-Sotos T, Prieto-Salceda D (2006). Cervical cancer mortality is increasing in Spanish women younger than 50. Cancer Lett.

[15] Anorlu RI (2008). Cervical cancer: the sub-Saharan African perspective. Reprod Health Matters.

[16] Garland S, Park SN, Ngan HY, Frazer I, Tay EH, Chen CJ, Bhatla N, Pitts M, Shin HR, Konno R, Smith J (2008). The need for public education on HPV and cervical cancer prevention in Asia: opinions of experts at the AOGIN conference. Vaccine.

[17] Moher D, Shamseer L, Clarke M, Ghersi D, Liberati A, Petticrew M, Shekelle P, Stewart LA (2015). Preferred reporting items for systematic review and meta-analysis protocols (PRISMA-P) 2015 statement. Syst Rev.

[18] Chapter 11: Systematic Reviews—Introduction JBI Reviewer’s Manual—JBI GLOBAL WIKI [Internet]. [cited 2020 April 20]. Available from: https://wiki.joannabriggs.org/display/MANUAL/1.1+Introduction+to+JBI+Systematic+reviews

[19] Risk of Bias Instrument for Cross-Sectional Surveys of Attitudes and Practices [Internet]. CLARITY Group at McMaster University; [cited 14 May 2020]. Available from: https://www.evidencepartners.com/wp-content/uploads/2017/09/Risk-of-Bias-Instrument-for-Cross-Sectional-Surveys-of-Attitudes-and-Practices.pdf

[20] Abotchie PN, Shokar NK (2009). Cervical cancer screening among college students in Ghana: knowledge and health beliefs. Int J Gynecol Cancer.

[21] Agboeze J, Nwali M, Ezeani N (2018). Cervical cancer screening knowledge and behavior among female undergraduate students in a Nigerian University. J Glob Oncol.

[22] Akujobi CN, Ikechebelu JI, Onunkwo I, Onyiaorah IV (2008). Knowledge, attitude and practice of screening for cervical cancer among female students of a tertiary institution in South Eastern Nigeria. Niger J Clin Pract.

[23] Al-Naggar RA, Low WY, Isa ZM (2010). Knowledge and barriers towards cervical cancer screening among young women in Malaysia. Asian Pac J Cancer Prev.

[24] Al-Shaikh GK, Almussaed EM, Fayed AA, Khan FH, Syed SB, Al-Tamimi TN, Elmorshedy HN (2014). Knowledge of Saudi female university students regarding cervical cancer and acceptance of the human papilloma virus vaccine. Saudi Med J.

[25] Albuquerque CL, Costa MD, Nunes FM, Freitas RW, Azevedo PR, Fernandes JV, Rego JV, Barreto HM (2014). Knowledge, attitudes and practices regarding the Pap test among women in northeastern Brazil. Sao Paulo Med J.

[26] Alwahaibi NY, Alramadhani NM, Alzaabi AM, Alsalami WA (2017). Knowledge, attitude and practice of Pap smear among Omani women. Ann Trop Med Public Health.

[27] Annan FM, Asante KO, Kugbey N (2019). Perceived seriousness mediates the influence of cervical cancer knowledge on screening practices among female university students in Ghana. BMC Womens Health.

[28] Ayinde OA, Omigbodun AO, Ilesanmi AO (2004). Awareness of cervical cancer, Papanicolaou's smear and its utilisation among female undergraduates in Ibadan. Afr J Reprod Health.

[29] Bigaard J, Mortensen JH, Kvernrød AB (2018). Barriers for young women to participate in the Danish cervical screening program. J Global Oncol.

[30] Binka C, Nyarko SH, Doku DT (2016). Cervical cancer knowledge, perceptions and screening behaviour among female university students in Ghana. J Cancer Educ.

[31] Black AT, McCulloch A, Martin RE, Kan L. Young women and cervical cancer screening: what barriers persist? Can J Nurs Res Arch. 2011;8–21. 21661613

[32] Blomberg K, Tishelman C, Ternestedt BM, Törnberg S, Levál A, Widmark C (2011). How can young women be encouraged to attend cervical cancer screening? Suggestions from face-to-face and internet focus group discussions with 30-year-old women in Stockholm, Sweden. Acta Oncol.

[33] Blomberg K, Widmark C, Ternestedt BM, Törnberg S, Tishelman C (2011). Between youth and adulthood: focus group discussions with 30-year-old women about cervical cancer and its prevention in urban Sweden. Cancer Nurs.

[34] Byrd TL, Peterson SK, Chavez R, Heckert A (2004). Cervical cancer screening beliefs among young Hispanic women. Prev Med.

[35] Duffett-Leger LA, Letourneau NL, Croll JC (2008). Cervical cancer screening practices among university women. J Obstet Gynecol Neonatal Nurs.

[36] Head KJ, Cohen EL (2012). Young women’s perspectives on cervical cancer prevention in Appalachian Kentucky. Qual Health Res.

[37] Hobbs M (2000). Adolescents had poor knowledge about Papanicolaou (cervical) smear screening and identified many barriers to being screened. Evid Based Nurs.

[38] Hoque ME (2013). Awareness of cervical cancer, Papanicolau's smear and its utilization among female, final year undergraduates in Durban, South Africa. J Cancer Res Ther.

[39] Lorenzi NP, Termini L, Longatto Filho A, Tacla M, de Aguiar LM, Beldi MC, Ferreira-Filho ES, Baracat EC, Soares-Júnior JM (2019). Age-related acceptability of vaginal self-sampling in cervical cancer screening at two university hospitals: a pilot cross-sectional study. BMC Public Health.

[40] Jayasinghe Y, Rangiah C, Gorelik A, Ogilvie G, Wark JD, Hartley S, Garland SM (2016). Primary HPV DNA based cervical cancer screening at 25 years: Views of young Australian women aged 16–28 years. J Clin Virol.

[41] Jubelirer SJ, Blanton MF, Blanton PD, Zhang J, Foster D, Monk J, Kuhn G, Hanshew D (1996). Assessment of knowledge, attitudes, and behaviors relative to cervical cancer and the Pap smear among adolescent girls in West Virginia. J Cancer Educ.

[42] Kahn JA, Chiou V, Allen JD, Goodman E, Perlman SE, Emans SJ (1999). Beliefs about Papanicolaou smears and compliance with Papanicolaou smear follow-up in adolescents. Arch Pediatr Adolesc Med.

[43] Kaneko N (2018). Factors associated with cervical cancer screening among young unmarried Japanese women: results from an internet-based survey. BMC Womens Health.

[44] Kim HW (2014). Factors related to the perceptions of susceptibility and severity in cervix cancer among unmarried university women in Korea. J Women’s Health.

[45] Kim HW (2019). Attitudetoward the PAP test among the sexually active unmarried women. J Women’s Health.

[46] Langille DB, Rigby JA (2006). Factors associated with PAP testing in adolescents in northern Nova Scotia. Can J Public Health.

[47] Lee HY, Lee MH (2017). Barriers to cervical cancer screening and prevention in young Korean immigrant women: implications for intervention development. J Transcult Nurs.

[48] Najem GR, Batuman F, Smith AM (1996). Papanicolaou test status among inner-city adolescent girls. Am J Prev Med.

[49] Ogbonna FS (2017). Knowledge, attitude, and experience of cervical cancer and screening among Sub-saharan African female students in a UK University. Ann Afr Med.

[50] Okoeki MO, Steven A, Geddes L (2016). Psychological factors affecting participation in cervical screening for young women: a qualitative study. Lancet.

[51] Oshima S, Maezawa M (2013). Perception of cervical cancer screening among Japanese university students who have never had a pap smear: a qualitative study. Asian Pac J Cancer Prev.

[52] Pan XF, Zhao ZM, Sun J, Chen F, Wen QL, Liu K, Song GQ, Zhang JJ, Wen Y, Fu CJ, Yang CX (2014). Acceptability and correlates of primary and secondary prevention of cervical cancer among medical students in southwest China: implications for cancer education. PLoS ONE.

[53] Waller J, Jackowska M, Marlow L, Wardle J (2012). Exploring age differences in reasons for nonattendance for cervical screening: a qualitative study. BJOG Int J Obstetr Gynaecol.

[54] Kahn JA, Goodman E, Slap GB, Huang B, Emans SJ (2001). Intention to return for Papanicolaou smears in adolescent girls and young women. Pediatrics.

[55] Hoque ME, Ghuman S, Coopoosmay R, Van Hal G (2014). Cervical cancer screening among university students in South Africa: use of health belief model. Int J Infect Dis.

[56] Glauser W. Primary care system outdated and inconvenient for many millennials. 2018. E1430–E1431. Available at https://www.cmaj.ca/content/190/48/E1430.short. 10.1503/cmaj.109-5688 PMC6258217 30510054

[57] Nabalamba, A., and W. Millar. 2007. “Going to the doctor”. Health Reports. Statistics Canada Catalogue no. 82–003. Vol. 18 no. 1. (accessed January 11, 2017) 17441441

[58] Black WC, Nease Jr RF, Tosteson AN. Perceptions of breast cancer risk and screening effectiveness in women younger than 50 years of age. J Nat Cancer Inst. 1995;87(10):720–731. 10.1093/jnci/87.10.720 7563149

[59] Lam AC, Aggarwal R, Cheung S, Stewart EL, Darling G, Lam S, Xu W, Liu G, Kavanagh J. Predictors of participant nonadherence in lung cancer screening programs: a systematic review and meta-analysis. Lung Cancer. 2020. 10.1016/j.lungcan.2020.05.013 32535225

[60] Schootman M, Jeffe DB, Baker EA, Walker MS (2006). Effect of area poverty rate on cancer screening across US communities. J Epidemiol Community Health.

[61] Bennett KJ, Pumkam C, Bellinger JD, Probst JC (2011). Cancer screening delivery in persistent poverty rural counties. J Primary Care Community Health.

[62] Hammond WP (2010). Psychosocial correlates of medical mistrust among African American men. Am J Community Psychol.

[63] Arnett MJ, Thorpe RJ, Gaskin DJ, Bowie JV, LaVeist TA (2016). Race, medical mistrust, and segregation in primary care as usual source of care: findings from the exploring health disparities in integrated communities study. J Urban Health.

[64] McDonald JT, Kennedy S (2004). Insights into the ‘healthy immigrant effect’: health status and health service use of immigrants to Canada. Soc Sci Med.

[65] Kennedy S, Kidd MP, McDonald JT, Biddle N. The healthy immigrant effect: patterns and evidence from four countries. J Int Migr Integr. 2015;16(2):317–32.

[66] Barnholtz-Sloan J, Patel N, Rollison D, Kortepeter K, MacKinnon J, Giuliano A (2009). Incidence trends of invasive cervical cancer in the United States by combined race and ethnicity. Cancer Causes Control.

[67] Johnson CE, Mues KE, Mayne SL, Kiblawi AN (2008). Cervical cancer screening among immigrants and ethnic minorities: a systematic review using the Health Belief Model. J Low Genit Tract Dis.

[68] Ferris DG, Batish S, Wright TC, Cushing C, Scott EH (1996). A neglected lesbian health concern: cervical neoplasia. J Fam Pract.

[69] Clark MA, Boehmer U, Rosenthal S. Cancer screening in lesbian and bisexual women and trans men. In: Cancer and the LGBT Community 2015 (pp. 83–98). Springer, Cham.

[70] Tracy JK, Lydecker AD, Ireland L (2010). Barriers to cervical cancer screening among lesbians. J Womens Health.

[71] Johnson M, Wakefield C, Garthe K (2020). Qualitative socioecological factors of cervical cancer screening use among transgender men. Prev Med Rep.

[72] Sundstrom B, Smith E, Delay C, Luque JS, Davila C, Feder B, Paddock V, Poudrier J, Pierce JY, Brandt HM (2019). A reproductive justice approach to understanding women's experiences with HPV and cervical cancer prevention. Soc Sci Med.

[73] Luszczynska A, Durawa AB, Scholz U, Knoll N (2012). Empowerment beliefs and intention to uptake cervical cancer screening: three psychosocial mediating mechanisms. Women Health.

